# Analyzing Morphology, Metabolomics, and Transcriptomics Offers Invaluable Insights into the Mechanisms of Pigment Accumulation in the Diverse-Colored Labellum Tissues of *Alpinia*

**DOI:** 10.3390/plants12213766

**Published:** 2023-11-03

**Authors:** Tong Zhao, Qianxia Yu, Canjia Lin, Huanfang Liu, Limei Dong, Xinxin Feng, Jingping Liao

**Affiliations:** 1 Guangdong Eco-engineering Polytechnic, Guangzhou 510520, China; 18825183218@163.com; 2 Guangdong Provincial Key Laboratory of Crop Genetic Improvement, South China Peanut Sub-Center of National Center of Oilseed Crops Improvement, Crops Research Institute, Guangdong Academy of Agricultural Sciences, Guangzhou 510640, China; yuqianxia@gdaas.cn; 3 Key Laboratory of Plant Resources Conservation and Sustainable Utilization, South China Botanical Garden, Chinese Academy of Sciences, Guangzhou 510650, China; lincanjia@scbg.ac.cn (C.L.); hfliu@scbg.ac.cn (H.L.); liaojp@scbg.ac.cn (J.L.); 4 Dongguan Botanical Garden, Dongguan 523086, China; xinxin8715@163.com

**Keywords:** *Alpinia*, pigment accumulation, flavonoids, carotenoids, transcriptome sequencing, metabolome

## Abstract

*Alpinia* plants are widely cherished for their vibrant and captivating flowers. The unique feature of this genus lies in their labellum, a specialized floral structure resulting from the fusion of two non-fertile staminodes. However, the intricate process of pigment formation, leading to distinct color patterns in the various labellum segments of *Alpinia*, remains a subject of limited understanding. In this study, labellum tissues of two *Alpinia* species, *A. zerumbet* (yellow–orange flowers) and *A. oxyphylla* (white–purple flowers), were sampled and analyzed through morphological structure observation, metabolite analysis, and transcriptome analyses. We found that hemispherical/spherical epidermal cells and undulate cell population morphology usually display darker flower colors, while flat epidermal cells and cell populations usually exhibit lighter flower colors. Metabolomic analysis identified a high concentration of anthocyanins, particularly peonidin derivatives, in segments with orange and purple pigments. Additionally, segments with yellow pigments showed significant accumulations of flavones, flavanols, flavanones, and xanthophylls. Furthermore, our investigation into gene expression levels through qRT-PCR revealed notable differences in several genes that participated in anthocyanin and carotenoid biosynthesis among the four pigmented segments. Collectively, these findings offer a comprehensive understanding of pigmentation in *Alpinia* flowers and serve as a valuable resource for guiding future breeding efforts aimed at developing *Alpinia* varieties with novel flower colors.

## 1. Introduction

**Figure 1:**
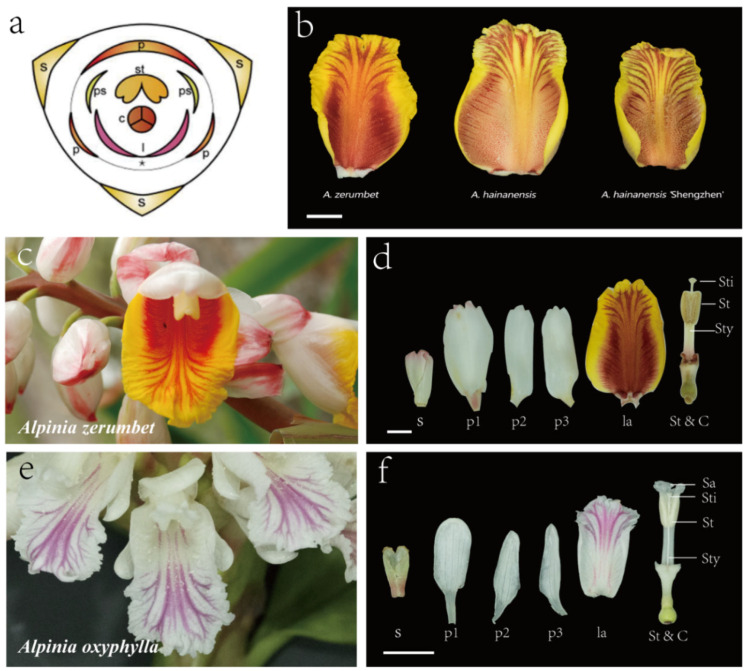
Floral morphology of *Alpinia* flowers. (**a**) Floral diagram of Zingiberaceae; (**b**) Labellum in *Alpinia zerumbet*, *A. hainanensis* and *A. hainanensis* ‘Shengzhen’; (**c**,**d**) Flower morphology and flower organ anatomy of *A. zerumbet*; (**e**,**f**) Flower morphology and flower organ anatomy of *A. oxyphylla*; S sepal, p1, p2, p3 petal, ps petaloid staminode, st stamen, l/la labellum, C carpel, sti stigma, sty style, sa stamen appendage. The asterisk indicates the degenerated abaxial staminode.

Zingiberales constitute a monophyletic order with two main groups: the “ginger group”, which encompasses Marantaceae, Cannaceae, Zingiberaceae, and Costaceae, and the “banana group”, which includes Strelitziaceae, Musaceae, Lowiaceae, and Heliconiaceae [1,2,3]. Within the ginger group, all four subgroups share a characteristic feature where petaloid staminodes replace the majority stamens in mature flowers [4,5]. *Alpinia*, a genus belonging to the Zingiberaceae family, stands out as a group of plants with substantial ornamental and medicinal significance. The flowers of this genus consist of four distinct floral whorls (Figure 1a): the outer perianth comprises three sepals, followed by three petals forming the inner perianth. The androecial whorl consists of one fertile stamen and five staminodes, with two of these typically fusing to develop a particular abaxial labellum. Another two adaxial staminodes degenerate into diamond-shaped appendages, persistent at the base of the labellum. The last abaxial staminode degenerates and disappears. The innermost whorl features a tricarpellary pistil. In most flowering species, stamens primarily serve reproductive functions and do not contribute to the visual appeal of the flower. *Alpinia*, however, presents an exception, as the perianth is inconspicuous in mature flowers and constitutes only some components of the floral display. Instead, the labellum that is formed by the fusion of two petaloid staminodes occupies a position homologous to stamens but exhibits properties more akin to perianth than androecium.

The color and pattern of flowers, as key visual indicators of flower organ identity, are widely regarded as the most crucial ornamental characteristics. In the case of *Alpinia* flowers, the color pattern of the labellum stands out as the most distinctive feature, directly influencing both aesthetic appeal and commercial value. Variations in pigment distribution across various segments of the labellum give rise to the diverse flower color arrangements, encompassing an array of colors and stripes, that are characteristic of *Alpinia*. The color and stripe patterns of labella in *Alpinia* exhibit notable differences not only across various species but even within different varieties of the same species (Figure 1b). This distinctiveness often forms the morphological basis for distinguishing between different *Alpinia* species or varieties. For instance, *A. zerumbet*, characterized by its bright yellow labellum adorned with orange stripes (Figure 1c,d), and *A. oxyphylla*, displaying a white labellum with purple stripes (Figure 1e,f), are particularly esteemed ornamental plants for cultivation in tropical and subtropical regions. These two species represent a two-color series of labellum patterns within *Alpinia*, making them ideal candidates for investigating the pattern of pigment accumulation in the labellum of this genus. Despite numerous studies shedding light on the mechanisms of pigment biosynthesis in flowers [6,7,8], the intricacies of different types of pigments in labellum segments in *Alpinia* remain poorly understood. The exploration of these mechanisms underlying color differentiation in *Alpinia* labellum is of great importance. It not only enhances our understanding of flower evolution and diversification but also offers valuable insights for breeding novel ornamental varieties.

The colored compound distribution and accumulation in various floral segments are reported to be affected by three key factors: organizational structure, secondary metabolites, and gene expression regulation [9,10,11]. Previous research has revealed that the shapes of epidermal cells in petals can significantly impact visual perception by affecting light reflection [12,13]. For instance, in lilies, there is a notable difference in cell morphology between unpigmented and pigmented regions of petals [14]. Epidermal cells tend to flatten in the unpigmented regions, while in the pigmented areas, they appear embedded and convex [14]. In the epidermis of Primulaceae flowers, pigmented cells are predominantly circular, curved, or sharp cone shaped [15]. Furthermore, variations in accumulation of flavonoids, carotenoids and their derivatives play a pivotal role in determining floral color. Their presence and abundance contribute significantly to the overall pigmentation and color diversity in the plant kingdom. Flavonoids, as one of the primary pigment groups responsible for most flower colors, encompasses a broad spectrum from orange to blue (anthocyanins) and yellow (flavonols, chalcones, and aurones) [16]. Carotenoids, which belong to the C40 isoprenoid class and include carotenes and xanthophylls, contribute to distinct colors spanning yellow, orange, and red [17,18]. In most cases, anthocyanins and carotenoids coexist and combine to produce a wide range of diverse flower colors [16].

In ornamental plants, distinctive pigmentation patterns such as stripes or spots are often closely linked to the spatial gene expression. The enzymes pivotal to flavonoids and carotenoids biosynthesis have been extensively characterized, including chalcone synthase (CHS), chalcone isomerase (CHI), flavanone 3-hydroxylase (F3H), flavanone 3′ hydroxylase (F3′H), flavone synthase (FNS), flavonol synthase (FLS), dihydroflavonol 4-reductase (DFR), anthocyanidin synthase (ANS), phytoene synthase (PSY), phytoene desaturase (PDS), and ζ-carotene desaturase (ZDS) [9,10,19]. The spatiotemporal regulation of genes related to pigment biosynthesis is responsible for generating specific pigmentation patterns. Although anthocyanins’ and carotenoids’ biosynthetic pathways have been thoroughly investigated in numerous species, the dynamic expression of genes linked to anthocyanin and carotenoid biosynthesis during *Alpinia* labellum color formation warrants further investigation. The application of UPLC/ESI-Q TRAP-MS/MS, known for its advantage of rapid separation, exceptional sensitivity, extensive coverage, and high throughput, has found widespread use in identifying and analyzing metabolites in various plants, including tomato, strawberry, lily, and tree peony [14,20,21,22]. In this study, labellum tissues from two *Alpinia* species, *A. zerumbet* and *A. oxyphylla*, were sampled and analyzed through tissue structure, metabolomics, and gene expression. Our findings illuminate the key pigments and the associated biosynthesis genes that underlie the formation of labellum color in *Alpinia* flowers.

## 2. Results

### 2.1. Differences in Epidermal Cell Morphology in Four Pigmented Segments of Alpinia Labellum

**Figure 2:**
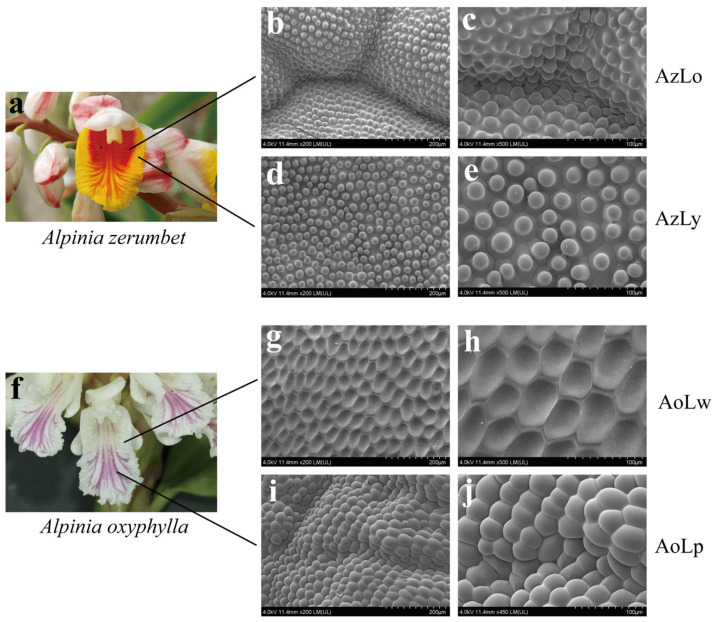
Cryoscanning electron microscopy examination of the epidermal cells of *Alpinia* labella. (**a**) Flower of *A. zerumbet*; (**b**–**e**) Epidermal cell morphology in different pigmented segments in *A. zerumbet*’s labellum, with 200× ((**b**,**d**), Bar = 200 μm) and 500× ((**c**,**e**), Bar = 100 μm) magnification; (**f**) Flower of *A. oxyphylla*; (**g**–**j**) Epidermal cell morphology in different pigmented segments in labellum of *A. oxyphylla* with 200× ((**g**,**i**), Bar = 200 μm) and 500× (**h**)/450× (**j**) (Bar = 100 μm) magnification.

Cryo-EM analysis revealed significant variations in the morphology of individual epidermal cells and cell populations across the four-color segments of labella. In *A. zerumbet*, the morphology of single epidermal cells in AzLo (Figure 2b,c) and AzLy (Figure 2d,e), is characterized by a hemispherical convex shape. In *A. oxyphylla* (Figure 2f), the single epidermal cell in the unpigmented segment (AoLw, Figure 2g,h) tended to be flat, while the individual epidermal cells in the purple segment (AoLp, Figure 2i,j) exhibited a spherical convex shape. Most notably, the prominent arches and grooves are visible in the epidermal tissues of the orange (Figure 2b) and purple segments (Figure 2i), suggesting the cell population in AzLo and AoLp are arranged in an undulating pattern. However, the morphology of the cell population in the yellow segment (AzLy, Figure 2d) and unpigmented segment (AoLw, Figure 2g) appeared flat and inlaid, suggesting that they are embedded within the surrounding tissue in a more uniform manner.

### 2.2. Flavonoid Metabolic Differences in the Four Pigmented Segments of Alpinia Labella

To profile the flavonoid metabolic changes of different pigmented segments of *Alpinia* labellum, the flavonoid metabolite differences in four types of pigmented region (AzLy, AzLo, AoLw, and AoLp) were detected. Samples for three replicates were collected from different plants to eliminate individual differences. The UPLC/ESI-Q TRAP-MS/MS data were further examined for metabolites that accumulated in different pigmented labellum segments (AzLo vs. AzLy, AoLw vs. AoLp). A total of 332 flavonoid metabolites were identified, including 2 anthocyanins (peonidin derives), 17 chalcones, 23 flavanols, 30 flavanones, 7 flavanonols, 90 flavones, 141 flavonols, 9 proanthocyanidins, 10 tannins and 3 other flavonoids.

**Figure 3:**
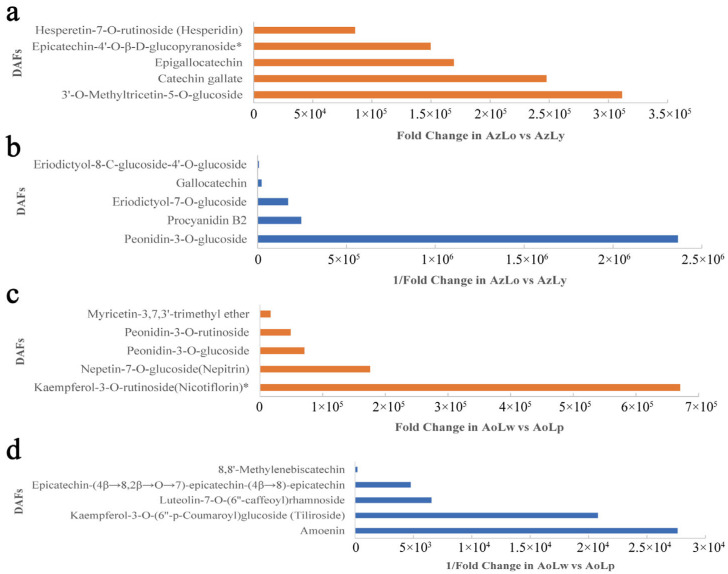
The top five upregulated and downregulated DAFs based on the fold change value. (**a**) The top five upregulated DAFs in AzLo vs. AzLy. (**b**) The top five downregulated DAFs in AzLo vs. AzLy. (**c**) The top five upregulated DAFs in AoLw vs. AoLp. (**d**) The top five downregulated DAFs in AoLw vs. AoLp. The asterisk in the upper right corner of the compound indicates that the compound was detected to have isomers.

The differentially accumulated flavonoids (DAFs) were identified based on the criterion of VIP ≥ 1 and |Log2FC| ≥ 1.0. A total of 190 and 130 DAFs were, respectively, found in AzLo vs. AzLy and AoLw vs. AoLp, of which 81 DAFs were shared by two comparison groups. Among all the 190 DAFs found in the AzLo vs. the AzLy group, 76 were downregulated and 114 were upregulated. The top 5 upregulated and downregulated DAFs were filtered based on the Fold Change (FC) value (shown in Figure 3). The most significantly upregulated metabolites (Figure 3a) were 3′-O-methyltricetin-5-O-glucoside, catechin gallate and epigallocatechin, indicating that these DAFs may be responsible for the accumulation of yellow pigments. Peonidin-3-O-glucoside, as one of the two anthocyanins detected in total flavonoids, displayed the most significant downregulation in AzLo vs. AzLy (Figure 3b), indicating that peonidin is more involved in pigment accumulation in the orange segment compared to the yellow segment. Flavonoid metabolites with |log_2_FC| > 6 in AzLo vs. AzLy can be found in Table S2. In AoLw vs. AoLp, 29 downregulated and 101 upregulated DAFs out of 130 flavonoid metabolites were identified. Notably, the purple segment contained more anthocyanins, flavones, flavanols and flavanones than the unpigmented segment. In particular, kaempferol-3-O-rutinoside (nicotiflorin), followed by nepetin-7-O-glucoside (nepitrin) and peonidin-3-O-glucoside were significantly more abundant in the purple segment (AoLp) than in the un-pigmented segment (AoLw) (Figure 3c), indicating that these flavonoids participate more in the accumulation of purple pigments. The contents of amoenin, kaempferol-3-O-(6″-p-Coumaroyl) glucoside (tiliroside) and luteolin-7-O-(6″-caffeoyl) rhamnoside is significantly lower in AoLp compared to AoLw (Figure 3d), suggesting that these metabolites may have less association with the formation of purple pigments. The flavonoid metabolites with absolute Log_2_FC values > 6 in AoLw vs. AoLp are provided in Table S3. The top 5 pathways that were the most markedly enriched among the DAFs in AzLo vs. AzLy and AoLw vs. AoLp based on KEGG pathway analyses are shown in Figure S1.

### 2.3. Carotenoid Metabolic Differences in the Four Pigmented Segments of Alpinia Labella

To gain a precise understanding of carotenoid accumulation, carotenoid metabolites in four pigmented segments were analyzed using UPLC and APCI-MS/MS. In total, 42 and 8 carotenoids were found in AzLo vs. AzLy and AoLw vs. AoLp, respectively. Lutein (112.71 μg/g), zeaxanthin (16.38 μg/g) and lutein palmitate (13.1 μg/g) are three substances found in the orange segment (AzLo) with a content greater than 10 μg/g. The yellow segment of *Alpinia* labellum contains six types of carotenoid substances with a content greater than 10 μg/g, including lutein (1443.33 μg/g), zeaxanthin (308.81 μg/g), lutein palmitate (78.78 μg/g), α-carotene (34.85 μg/g), lutein myristate (25.09 μg/g) and β-carotene (19.58 μg/g), prompting that they are likely critical in imparting the yellow color to *Alpinia* labellum. In the purple and unpigmented segments, the contents of all eight types of carotenoids detected were less than 2 μg/g.

**Figure 4:**
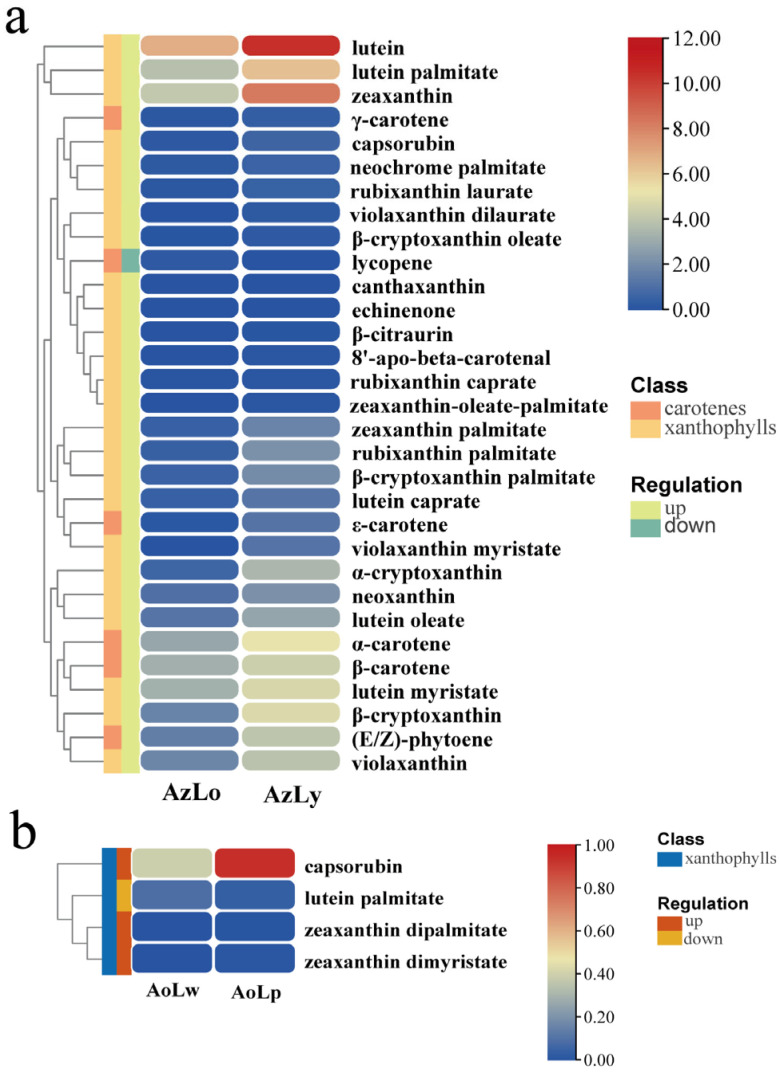
Heatmap showing the changes of DACs’ content in four colored segments of *Alpinia* labella. (**a**) The changes of DACs’ content in AzLo vs. AzLy. (**b**) The changes of DACs’ content in AoLw vs. AoLp.

Thirty-one differentially accumulated carotenoids (DACs) were found in AzLo vs. AzLy based on the identification criterion of |log2FoldChange| ≥ 2 or ≤ 0.5, of which one metabolite was downregulated, while 30 metabolites were upregulated (Figure 4a). Zeaxanthin, α-cryptoxanthin and lutein were the most significantly elevated metabolites, while lycopene was the only reduced metabolite (Figure 4a). It should be noted that violaxanthin dilaurate, violaxanthin myristate and β-citraurin were only identified in AzLy and the content of these three compounds were extremely low. In AoLw vs. AoLp, only four carotenoid metabolites were identified to be significantly different, including one downregulated metabolite (lutein palmitate) and three upregulated carotenoid metabolites (zeaxanthin dipalmitate, capsorubin and zeaxanthin dimyristate) (Figure 4b). The contents of these four DACs were less than 1 μg/g. The absence of significant carotenoid content in these segments suggests that other pigments may be responsible for their coloration.

### 2.4. Transcriptome Sequencing and Unigene Functional Annotation

Transcriptome sequencing of four distinct *Alpinia* labellum samples were analyzed to identify genes linked color formation. This effort yielded 885,596,366 raw reads of 2 × 150 bp from three biologically replicated RNA-seq libraries representing AzLo, AzLy, AoLw, and AoLp samples. Following the removal of poor-quality reads and adapters, 858,925,830 high-quality reads were subjected to assembly. This generated 184,458 unigenes, with a mean length of 1455 bp and an N50 length of 2123 bp. These genes were functionally annotated based on Nr, Swiss-prot, Pfam, KOG, and KEGG. A total of 126,414 unigenes were present in a minimum of one database. When assessing species similarity through Nr annotation, the highest similarity (70.46%, 87,060 unigenes) was observed with genes from Musa acuminata in the same order as *Alpinia*, followed by *Elaeis guineensis* (3.96%, 4888 unigenes) and *Phoenix dactylifera* (3.23%, 3991 unigenes). This pattern aligns with previous research conducted on *Zingiber zerumbet* and *Hedychium coronarium*, two species from the same family (Zingiberaceae) as *Alpinia* [23,24].

GO term analysis revealed that 107,970 unigenes were enriched in biological process, cellular component, and molecular function. Within the biological process, cellular process (70,005 unigenes, 64.8%) and metabolic process (55,852 unigenes, 51.7%) were the two largest subcategories. Specifically, 338, 27, 96, and 42 unigenes were identified in the flavonoid biosynthesis pathway (ko00941), anthocyanin biosynthesis pathway (ko00942), isoflavonoid biosynthesis pathway (ko00943), and flavone and flavonol biosynthesis pathway (ko00944), respectively. Additionally, 268 unigenes were related to carotenoid biosynthesis (ko00906) (Table S1).

### 2.5. Differentially Expressed Genes (DEGs) Involved in Flavonoid and Carotenoid Biosynthesis

Transcriptomic analyses comparing gene expression profiles between AzLo vs. AzLy and AoLw vs. AoLp to pinpoint key DEGs in differently colored segments of *Alpinia* labellum detected 12,840 DEGs meeting the criteria of a two-fold alteration at *p* < 0.05. Specifically, there were 5486 DEGs in the AzLo vs. AzLy comparison, comprising 2917 upregulated and 2569 downregulated genes (Figure S2a). The biological process included 51 flavonoid biosynthetic processes and 51 carotenoid biosynthetic processes (Figure S2b). A total of 7354 DEGs were identified in the AoLw vs. AoLp group, encompassing 3318 upregulated and 4036 downregulated genes (Figure S2c). The top 50 enriched GO pathways are displayed in Figure S2d. Comparing the results of KEGG enrichment analyses of DAFs and DEGs revealed six pathways that were enriched in both flavonoid metabolome and transcriptome. In the AzLo vs. AzLy group, these included metabolic pathways (7 metabolites, 1150 genes), biosynthesis of secondary metabolites (11 metabolites, 687 genes), flavonoid biosynthesis (11 metabolites, 36 genes), isoflavonoid biosynthesis (1 metabolite, 15 genes), flavone and flavonol biosynthesis (11 metabolites, 9 genes), and anthocyanin biosynthesis (1 metabolite, 1 gene). In the AoLw vs. AoLp group, metabolic pathways (10 metabolites, 1150 genes) and biosynthesis of secondary metabolites (14 metabolites, 642 genes) were the most enriched, followed by flavonoid biosynthesis (14 metabolites, 24 genes), isoflavonoid biosynthesis (1 metabolite, 8 genes), anthocyanin biosynthesis (1 metabolite, 3 genes), and flavone and flavonol biosynthesis (12 metabolites, 3 genes). Based on the joint analysis of the carotenoid metabolome and transcriptome data, four KEGG pathways that were enriched in both metabolome and transcriptome were identified. In AzLo vs. AzLy, these pathways were metabolic pathways (11 metabolites, 1150 genes), biosynthesis of secondary metabolites (14 metabolites, 687 genes), biosynthesis of cofactors (2 metabolites, 122 genes), and carotenoid biosynthesis (15 metabolite, 53 gene). In AoLw vs. AoLp, only carotenoid biosynthesis was shared (1 metabolites, 15 genes). These pathways and genes enriched by both the metabolome and transcriptome provide us with directions for screening potential genes involved in flavonoid and carotenoid biosynthesis.

**Figure 5:**
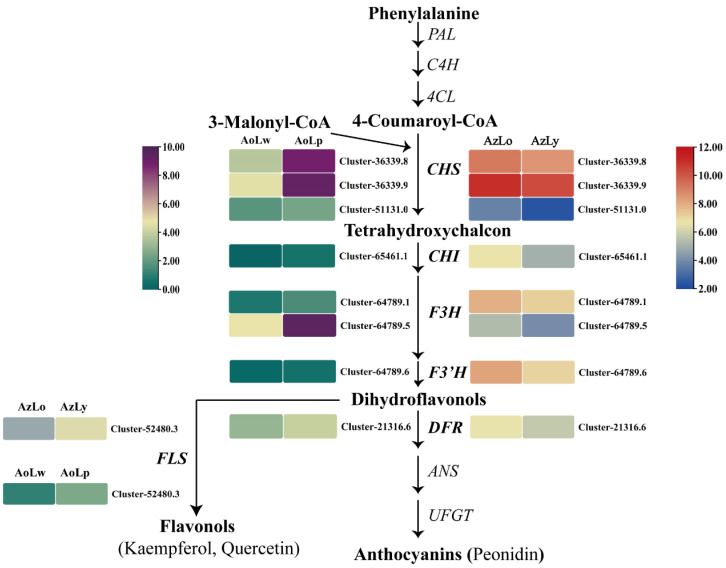
Putative genes linked to the flavonoid biosynthesis and their expression in four pigmented segments of labellum in *A. zerumbet* and *A. oxyphylla*. Each gene’s expression pattern is displayed in a heatmap alongside each step of the pathway. The RPKM values are presented with a color scale indicating the log-transformed RPKM value. Blue and green represent low expression in AzLo vs. AzLy and AoLw vs. AoLp, respectively. Red and purple represent high expression in AzLo vs. AzLy and AoLw vs. AoLp, respectively.

Analyses of unigenes involved in flavonoids and carotenoids, with particular focus on the anthocyanin and carotenoid biosynthesis pathway, identified DEGs related to flavonoid and carotenoid biosynthesis in AzLo vs. AzLy, including 4 structural genes for flavonoids (2 *CHSs*, 1 *CHI*, and 1 *DFR*), 5 *PSY*, 1 *PDS*, and 138 TF genes. DEGs linked to flavonoid and carotenoid biosynthesis were also identified in AoLw vs. AoLp, including 2 *CHSs*, 1 *DFR*, 1 *PSY*, 1 *PDS* and 173 TF genes. Figure 5 shows the putative genes linked to the flavonoid biosynthesis and their expression in four pigmented segments.

**Figure 6:**
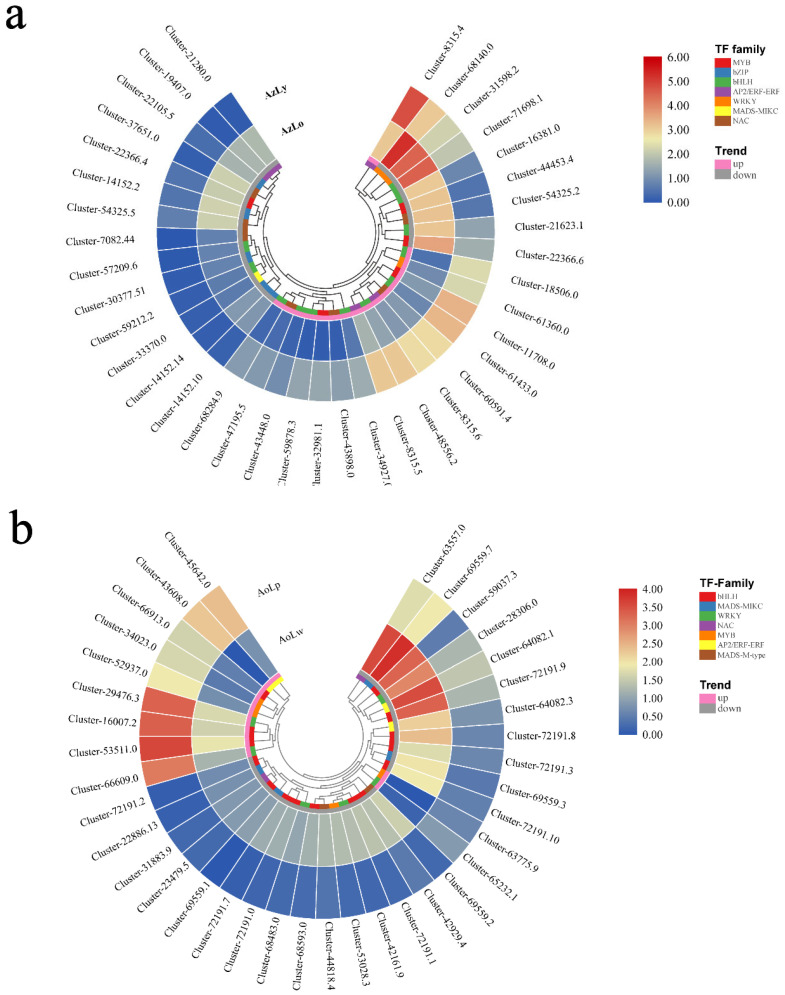
The expression pattern of differentially expressed transcription factors linked to flavonoid and carotenoid metabolite biosynthesis. TFs with an |log2FoldChange| > 2.0 were grouped based on a color-scaled log-transformed RPKM value. The color bars in the innermost circle indicate various TF families with corresponding colors representing their family identity (legend on the right). Pink and gray bars depict up and downregulated TFs, respectively. (**a**,**b**) show the expression pattern of differentially expressed TFs in AzLo (inner whorl) vs. AzLy (outer whorl) and in AoLw (inner whorl) vs. AoLp (outer whorl), respectively.

Among the transcription factors, 19 were annotated as MYB TFs, including MYB4, MYB44, and MYB86, MYB308. Twenty-one genes encoding bHLH and other TFs were also identified. The expression pattern of differentially expressed transcription factors with |log2FoldChange| > 2.0 are shown in Figure 6. These transcription factors probably play significant roles in flavonoid and carotenoid metabolite biosynthesis in *Alpinia* flowers.

### 2.6. Validation of Key Pigmentation-Related Gene Expression

**Figure 7:**
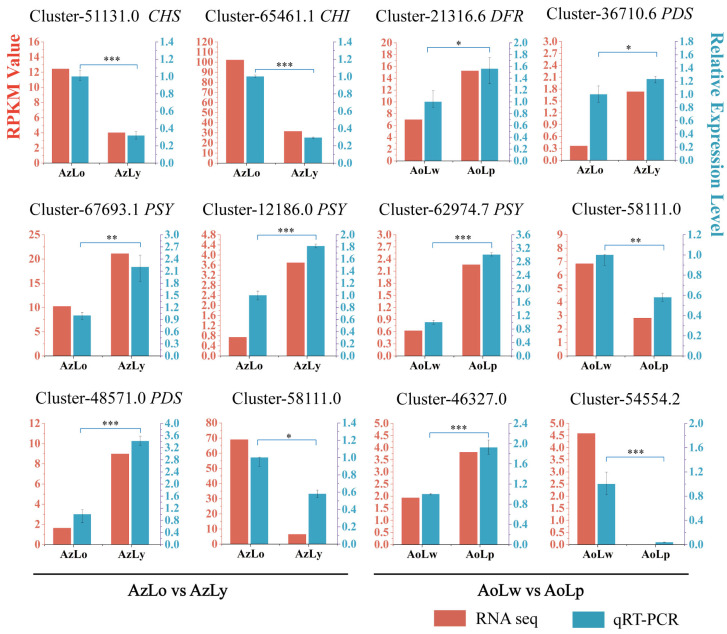
Relative expression of twelve key genes linked to pigmentation. Red and blue bars show each gene’s RPKM value by RNA-Seq analysis and relative expression by qRT-PCR, with relative level in AzLo and AoLw set to 1. The candidate gene names were listed behind the gene ID. Error bars represent standard deviation. The asterisk represents the statistical significance analyses of qRT-PCR.

Twelve unigenes were chosen to confirm the precision and reproducibility of the transcriptome analyses using qRT-PCR. Among them, eight unigenes were related to flavonoid and carotenoid biosynthetic pathways, and four were chosen for comparison. qRT-PCR analysis of their expression patterns was generally in line with their RPKM values from transcriptome analysis (Figure 7). In *A. zerumbet*, the expressions of *CHS* (Cluster-51131.0) and *CHI* (Cluster-65461.1) putative genes showed higher expression in AzLo than that in AzLy, while two *PSY*s (Cluster-67693.1 and Cluster-12186.0) and *PDS* (Cluster-48571.0) were significantly upregulated in the yellow segment (AzLy). In *A. oxyphylla*, the expression of *DFR* (Cluster-21316.6), *PDS* (Cluster-36710.6) and *PSY* (Cluster-62974.7) displayed a significant increase in AoLw vs. AoLp.

## 3. Discussion

### 3.1. Possible Correlation between the Various Flower Color Displays of Alpinia Labellum with the Morphology of Individual Epidermal Cells and Cell Populations

Previous studies have demonstrated that petal epidermal cell morphology has a great impact on light focusing [25,26,27], causing the actual color we see to be not a direct reaction to intracellular pigments. When light strikes the petals, it is partially absorbed by the epidermal cells and converted into energy. The remaining portion of the incident light is reflected back by the different organizational structures. For example, conical epidermal cells could capture more incident light, thereby increasing light absorption and promoting darker floral color development. However, flat epidermal cells tend to reflect more incident light, resulting in lighter floral color [7,28,29]. In *Alpinia*, the labellum, as a prominent organ in the overall floral display, is believed to perform a role similar to that of perianth whorls in attracting pollinators and providing aesthetic value, ultimately promoting reproduction and seed production. In this study, we noted substantial variations in epidermal cell morphology across various colored segments of *Alpinia* labella. From the perspective of cell population morphology, the epidermal cell population in orange (AzLo) and purple (AoLp) segments are undulatingly arranged, while the epidermal cell population in the yellow region (AzLy) and unpigmented region (AoLw) tend to be arranged in a flat pattern. From the perspective of individual cell morphology, the epidermal cells in the pigmented region of *Alpinia* were hemispherical (like AzLo and AzLy) or a spherical convex shape (like AoLp). These two organizational structures with irregular shapes and densely packed arrangements can reduce the amount of light reflected back and enhance light absorption from a physics perspective. In addition, the presence of pigments in the epidermal cells, such as anthocyanidins, flavonoids and carotenoids, can also contribute to light absorption. These pigments have specific molecular structures that allow them to absorb certain wavelengths of light, thereby reducing the overall reflection and giving rise to the observed coloration. In contrast, flat epidermal cells in AoLw have a smooth surface that allows light to reflect more easily. When light strikes these cells, a significant portion of it is reflected back without being absorbed. This reflection contributes to the perception of a lighter color or even white appearance in that particular area. Similar observations have been found for organizational structures of epidermal cells in the petals of lily cultivars [14]. This micro-morphological evidence demonstrates that the labellum of *Alpinia*, which is essentially composed of two petaloid staminodes, has similar features of organizational structures to that of petals. Therefore, the difference in the structure of epidermal cells and cell populations between the pigmented and unpigmented segments can lead to variations in the proportion of light reflection and absorption, ultimately influencing the color appearance in different segments of *Alpinia* labella.

### 3.2. The Differential Accumulation of Flavonoids and Anthocyanins in Different Color Segments Has a Significant Impact on the Color Formation of Alpinia Labella

Flavonoids are a class of secondary metabolites that play a critical role in the pigmentation processes of different plants. These compounds are responsible for the wide range of colors observed in flowers, fruits, and other plant tissues. Flavonoids, such as anthocyanins, flavones, flavonols, and proanthocyanidins, contribute to the red, purple, blue, and yellow colors in plants. Anthocyanins in particular, are important pigments responsible for red, purple, and blue colors in plants, and are synthesized through the flavonoid biosynthesis pathway. In *Alpinia*, our analysis of the flavonoid metabolome data unveiled a significant accumulation of anthocyanin compounds in the pigmented regions of the labellum, while comparatively lower levels were detected in the unpigmented area. Peonidin-3-O-rutinoside and peonidin-3-O-glucoside are two main metabolites differentially accumulated in anthocyanins in *Alpinia* labellum. In the AzLo vs. AzLy group, although peonidin-3-O-rutinosis is highly detected in both orange (AzLo) and yellow segments (AzLy), the content in AzLo was significantly higher than in AzLy, showing an almost 100-fold change. This suggests that peonidin-3-O-rutinoside may play a more prominent role in the orange pigmentation of AzLo. Additionally, peonidin-3-O-glucoside was found to be highly accumulated in the orange region (AzLo), but little accumulated in the yellow region (AzLy). This indicates that peonidin-3-O-glucoside may be specifically associated with the orange coloration in AzLo and may not contribute significantly to the yellow pigmentation in AzLy. These findings highlight the importance peonidin derives in determining the color differences between the orange and yellow segments in *Alpinia*. In the AoLw vs. AoLp group, these two anthocyanin substances are detected primarily in the purple segment, and are barely present in unpigmented segment, suggesting that these anthocyanins are specifically associated with the purple pigmentation observed in AoLp.

The levels of flavones (apigenin, luteolin, chrysin), flavanols (kaempferol, quercetin, epicatechin, catechin, gallocatechin), and flavanones (naringenin, hesperetin, dihydrochalcone) are elevated in the yellow segment compared to the orange segment. The specific enrichment of flavones, flavanols, and flavanones in the yellow segment indicates their involvement in the formation of yellow pigments in the labellum. The DAFs selection based on the fold change (FC) value, highlights the roles of specific metabolites, such as 3′-O-methyltricetin-5-O-glucoside, catechin gallate, epigallocatechin, and peonidin-3-O-glucoside, in the pigmentation processes and color differences between the orange and yellow segments. In addition, a small amount of anthocyanin components was detected in the white segment, suggesting the presence of unobservable pigments in the white segment. This could be due to the fact that fewer pigment substances are insufficient to generate visible color. Furthermore, the flat epidermal cells in unpigmented structures reflect more incident light, resulting in the absence of visible color in the unpigmented region. It is worth noting that the absence of visible color in the unpigmented segment does not necessarily mean that there are no pigments present. It is possible that other pigments, such as colorless flavonoids or carotenoids, may be present in the unpigmented region but are not visible to observe.

The regulation of gene expression and enzymatic activities involved in flavonoid biosynthesis ultimately determines the type and content of flavonoids produced in different plant tissues. In accordance with the changes in anthocyanin content, the higher expression levels of *CHS* and *CHI* in the orange segment (AzLo) compared to the yellow segment (AzLy) indicate their involvement in controlling flavonoid and anthocyanin metabolism. Furthermore, the upregulation of *DFR* in AoLp (purple segment) suggests its role in catalyzing the conversion of dihydroflavonols to colorless anthocyanins. This indicates that anthocyanins contribute more to the formation of orange and purple pigments in *Alpinia*, compared to the formation of the yellow pigment. Similar observations have been reported in two tree peony cultivars, where the reduction in anthocyanin content during flowering led to the transition in flower color from red to orange, and then to yellow [30]. Additionally, the decline in anthocyanins in two herbaceous peony cultivars resulted in elevated brightness and reduced redness, changing flowers from coral to pink and then to yellow [31]. Our results have uncovered that within the flavonoid biosynthesis pathway, the metabolites predominantly flow towards anthocyanin in the orange and purple segments of the labellum. Conversely, in the yellow segments, these metabolites are more directed towards the synthesis of flavones, flavanols and flavanones.

### 3.3. Carotenoid Biosynthesis Mainly Contributes to the Yellow Pigmentation of Alpinia Labellum

Carotenoids have been well-documented as key contributors to petal colors spanning from yellow to red in various flowering plants [17]. Several plant lineages featuring yellow flowers have been identified to harbor pigments originating from carotenoids [32]. α-carotene and β-carotene are carotenes that contribute to the yellow color in plants. Lutein, zeaxanthin, and lutein palmitate are xanthophylls known for their antioxidant properties and are commonly found in yellow and orange fruits and vegetables. Prior investigations in *Lonicera japonica* unveiled an increase in carotenoid quantity from the white flower to the yellow flower stages [33,34]. In *Alpinia*, the total content of carotenoids, including carotenes (ε-carotene and α-carotene) and xanthophylls (lutein, zeaxanthin etc.) in AzLy was significantly higher than in AzLo, indicating carotenoids are one of the main factors contributing to the color difference between the orange and yellow segments. Although lutein, zeaxanthin and lutein palmitate are the three carotenoids with the highest content in both the orange and yellow segments, the content of these three substances in the yellow segment are significantly higher than that in the orange segment (greater than ten-fold change), indicating these three substances contribute to the vibrant yellow color observed in *Alpinia* labellum. Lycopene, a carotenoid typically associated with red-colored plants, can only be detected in the orange segment, but the content is extremely low. The small amounts of lycopene present in the orange segment does not contribute significantly to the overall color difference between the orange and yellow segment. In the AoLw vs. AoLp comparison, only four carotenoids were identified, with zeaxanthin dipalmitate and zeaxanthin dimyristate exclusively detected in AoLp. Notably, these four types of carotenoids in AoLp were present in very low amounts (less than 2 μg/g), suggesting that carotenoids contribute little to the accumulation of purple pigments. Combined with the results of flavonoid metabolomics, it can be inferred that the formation of purple pigments in *Alpinia* labellum is mainly influenced by peonidin-3-O-rutinoside and peonidin-3-O-glucoside.

In the process of carotenoid biosynthesis, *PSY* plays a crucial role in catalyzing the condensation of two geranylgeranyl diphosphate (GGPP) molecules to form phytoene, and thereby enhance carotenoid accumulation in plants [35,36,37]. Subsequently, phytoene undergoes a series of desaturation reactions catalyzed by carotene desaturases, such as *PDS* and *ZDS* to form various carotenoids with different double bond configurations [18,36]. Similar observations have been reported in *Lilium* [38]. The petal colors of different cultivars of Asiatic hybrid lily have been found to be closely linked with the transcription levels of carotenoid biosynthetic genes, including *PSY*, *PDS*, and *ZDS* [38]. In *Alpinia*, *PSY* and *PDS* exhibited significantly higher expression levels in the yellow segment compared to the orange segment, which is in alignment with the variations in carotenoid content.

## 4. Materials and Methods

### 4.1. Plant Materials

*A. zerumbet* and *A. oxyphylla* grown at the South China Botanical Garden, Chinese Academy of Sciences, Guangzhou, China, were used in the study. Fresh flowers were collected from March to May in 2022. Four pigmented segments with three biological repeats were gathered from the labella of *Alpinia*, cryopreserved in liquid nitrogen for a minimum of 30 min and maintained at −80 °C for further RNA and metabolite extraction.

### 4.2. Cryo-Scanning Electron Microscopy

Samples were inspected with a cryo-scanning electron microscope (Cryo-SEM) (Hitachi SU8100, Tokyo, Japan) equipped with a temperature controller (PP3010T; Quoyum, East Sussex, UK). They were placed on a cryo-specimen holder and cryopreserved by immersion in slush nitrogen (−210 °C). Subsequently, they were swiftly moved to the cryo-unit while maintaining their frozen state. In the vacuum SEM chamber, the sample was sublimated at −90 °C. The examination was conducted with the cryo-stage SEM at an accelerating voltage of 1.0 keV. After successful sublimation, samples were sputtered with platinum and transferred to the SEM for direct viewing while being held at −145 °C. Imaging was achieved by capturing the secondary electron signal using a sensitive detector.

### 4.3. Transcriptome Sequencing

Total RNA was isolated from labellum samples AzLo, AzLy, AoLw and AoLp using RNA Plant Plus (TIANGEN, Beijing, China) per manufacturer’s recommendation and subjected to quality and quantity assessment using agarose gel electrophoresis, RNA Nano 6000 Assay (Agilent Technologies, Santa Clara, CA, USA), NanoPhotometer^®^ spectrophotometer (IMPLEN, Westlake Village, CA, USA) and Qubit^®^ RNA Assay (Life Technologies, Carlsbad, CA, USA). mRNA was isolated from qualified total RNA samples using oligo(dT), fragmented with divalent cations at elevated temperature, and converted into cDNA with random hexamer primers followed by second strand cDNA synthesis with DNA Polymerase I and RNase H. After purification using a QiaQuick PCR extraction kit (Qiagen, Venlo, The Netherlands), double-stranded cDNA samples were end repaired [39]. The libraries were prepared by adding Illumina sequencing adapters, size selection and PCR amplification and sequenced on Illumina platform to obtain 150 bp pair-end reads. High-quality reads were obtained after removal of adapters, uncertain nucleotides and low-quality sequences and de novo assembled using Trinity v2.4.0 [40].

### 4.4. Gene Functional Annotation and Differential Expression

Gene functions were annotated with E-value of 1 × 10^−5^ to NR as the threshold using BLASTx (https://www.ncbi.nlm.nih.gov, accessed on 30 May 2022), Swiss-Prot (http://www.uniprot.org/, accessed on 30 May 2022), Pfam (http://pfam.xfam.org/, accessed on 30 May 2022), Clusters of Orthologous Groups of proteins (KOG, ftp://ftp.ncbi.nih.gov/pub/COG/COG, accessed on 30 May 2022) and Kyoto Encyclopedia of Genes and Genomes (KEGG, http://www.genome.jp/eg/, accessed on 30 May 2022). Gene Ontology (GO) annotation was analyzed by the Blast2GO 2.3.5 program based on Nr annotation [41]. Candidate CDS were predicted using TransDecoder (https://github.com/TransDecoder/TransDecoder/wiki, accessed on 30 May 2022). Transcription factors were analyzed using iTAK.

The relative levels of each unigene were defined as reads per kilobase per million mapped reads (RPKM) [42]. Genes with |log2FoldChange| ≥ 2 and a false discovery rate (FDR) < 0.05 were detected using edgeR 3.12.1 [43] as differentially expressed genes (DEG). The heatmap of differentially expressed transcription factors was conducted with TBtools [44].

### 4.5. Real-Time Quantitative PCR (qRT-PCR) for RNA Validation

Expression of twelve unigenes were validated using qRT-PCR. Primers were selected using Integrated DNA Technologies PrimerQuest tool (https://sg.idtdna.com/primerquest/home/index, accessed on 15 September 2022) and can be found in Table S4. qRT-PCR was conducted in 20 μL of solution comprising 10 μL of Hieff^®^ qPCR SYBR Green Master Mix (No Rox) (Yeasen Biotech, Shanghai, China), 0.5 μL of 10 μmol/L each primer, and 4 μL of template with three technical replicates on a LongGene Q2000B Real-time PCR Detection system (LongGene Instruments, Hangzhou, China) at 95 °C for 3 min before 40 cycles of 10s at 95 °C and 30 s at 60 °C. Transcript levels relative to β-actin were measured using the 2^−∆∆Ct^ method [45] and plotted using Origin 9.0. The statistical significance analyses were conducted through independent-samples *t*-test using SPSS Statistics 17.

### 4.6. Flavonoid Metabolite Isolation and Analysis

The freeze-dried labella were pulverized using a mixer mill (MM 400, Retsch, Westfalen, Germany) with a zirconia bead at 30 Hz for 1.5 min. Subsequently, 50 mg of samples were resuspended in 1.2 mL of 70% methanol by vortexing for 30 s every 30 min for a total of 6 times and spun at 12,000 rpm for 3 min. The supernatants were passed through a 0.22 μm filter (SCAA-104; ANPEL, Shanghai, China). For UPLC-MS/MS, 4 μL of filtrate was injected onto an Agilent SB-C18 column (1.8 µm, 2.1 mm × 100 mm) and separated at 40 °C with 0.1% formic acid water and acetonitrile solution at a gradient program of 95:5 at 0 min, 5:95 at 9 min, 5:95 at 10 min, 95:5 at 11.1 min, and 95:5 at 14 min at 0.35 mL/min. Each fraction was subjected in turn to ESI-triple quadrupole linear ion trap mass spectrometry analysis with source temperature at 550 °C, ion spray voltage at 5500 V (positive ion mode)/−4500 V (negative ion mode), ion source gas I at 50.0 psi, ion source gas II at 60.0 psi, curtain gas at 25 psi, and the collision-activated dissociation at high. The instrument was tuned in QQQ mode with 10 μmol/L polypropylene glycol solutions, and mass was calibrated in LIT mode with 100 μmol/L polypropylene glycol solutions. Multiple reaction monitoring experiments were employed for QQQ scans with medium collision nitrogen gas. Optimal de-clustering potential and collision energy for each MRM transition were achieved by fine-tuning additional DP and CE settings. During each duration, a distinct MRM transition group was tracked to corresponding metabolites within that time window.

### 4.7. Carotenoids, Metabolite Isolation and Analysis

The sample underwent freeze-drying, followed by grinding into a powder (30 Hz, 1.5 min), and was subsequently kept at −80 °C. A 50 mg portion was accurately measured, resuspended in 0.5 mL of mixture of n-hexane, acetone, and ethanol at 1:1:1 (*v*/*v*/*v*), vortexed at room temperature for 20 min, and centrifugated at 12,000 rpm for 5 min at 4 °C. After repeating the procedure once, the supernatants were combined, dried by evaporation, reconstituted in a MeOH/MTBE (1:1, *v*/*v*) solution, and passed through a 0.22 μm filter. The filtrate was then analyzed using LC-MS/MS as previously reported [46,47,48].

For analyzing carotenoid metabolomics with a UPLC-APCI-MS/MS system, 2 μL of the filtrates were injected onto a YMC C30 LC column (3 μm, 100 mm × 2.0 mm) and separated with a mobile phase of methanol: acetonitrile (1:3, *v*/*v* ratio) with 0.01% BHT and 0.1% formic acid and methyl tert-butyl ether with 0.01% BHT at a gradient of 100:0 *v*/*v* at 0 min, 30:70 *v*/*v* at 3 min, 5:95 *v*/*v* at 5 min, and 100:0 *v*/*v* at 10 min at a flow rate of 0.8 mL/min at 28 °C. The effluent was then directed to a QTRAP^®^ 6500+ LC-MS/MS system outfitted with an APCI heated nebulizer and analyzed in positive-ion mode using Analyst 1.6.3 software (Sciex). The APCI source operation parameters were set as follows: ion source: APCI+, source temperature at 350 °C and curtain gas at 25.0 psi. Carotenoids were detected with scheduled MRM with CAD as the medium. DP and CE were fine-tuned for each MRM transition, and the quantification of all metabolites was performed with Multiquant 3.0.3 software (Sciex).

### 4.8. Selection of Differential Metabolites

Differential flavonoid metabolomics were selected based on the thresholds of VIP ≥ 1 and |Log2FC| ≥ 1.0. The VIP value was obtained from OPLS-DA. The score and permutation plots were established with R package MetaboAnalystR. Before conducting OPLS-DA, the data underwent a log2 transformation and mean centering. A permutation test involving 200 permutations was executed to prevent overfitting.

## 5. Conclusions

Taken together, colors in different segments of *Alpinia* labellum were affected by organizational structure, the contents of flavonoids and carotenoids, and gene expression regulation. The structure of both individual epidermal cells and cell population morphology have influence on the visual effect of floral colors. Hemispherical/spherical epidermal cells and undulate cell population morphology usually displays darker flower colors, while flat epidermal cells and cell populations usually exhibit lighter flower colors. The color difference between orange and yellow is primarily shaped by the collaborative effects of flavonoids and carotenoids, with orange pigments mainly comprising anthocyanins and xanthophylls, and yellow pigments primarily influenced by flavones, flavanols, flavanones, xanthophylls and carotenes. The variation in color between white and purple is mainly influenced by the differential accumulation of anthocyanins. In addition, several genes linked to anthocyanin and carotenoid biosynthesis displayed significantly different expression levels among the four pigmented segments. These findings offer a comprehensive understanding of pigment accumulation in *Alpinia* flowers and function as an invaluable resource for future development of *Alpinia* varieties with novel flower colors.

## Data Availability

The RNA-seq data were deposited in the National Center for Biotechnology Information Short Read Archive (NCBI-SRA) database (https://www.ncbi.nlm.nih.gov/Traces/sra, accessed on 30 October 2023) under accession number PRJNA1033550.

## Supplementary Materials

The following supporting information can be downloaded at: https://www.mdpi.com/article/10.3390/plants12213766/s1, Table S1: KEGG pathways linked to flavonoid and carotenoid biosynthesis; Table S2: Differential flavonoid metabolites in AzLo vs. AzLy, asterisk indicates that the compound was detected to have isomers; Table S3: Differential flavonoid metabolites in AoLw vs. AoLp, asterisk indicates that the compound was detected to have isomers; Table S4: qRT-PCR primers; Figure S1: The top five KEGG pathways markedly enriched among the DAFs; Figure S2: Analysis of DEGs in labellum libraries of *A. zerumbet* and *A. oxyphylla*.
