# Supplementary material for: Analyzing Morphology, Metabolomics, and Transcriptomics Offers Invaluable Insights into the Mechanisms of Pigment Accumulation in the Diverse-Colored Labellum Tissues of Alpinia
Source: Plants (Basel). 2023 Nov 3;12(21):3766. doi: 10.3390/plants12213766 (PMC10650467; doi:10.3390/plants12213766)
Supplement: Supplementary file 1 [file Figure S1 and S2.pdf › Figure S1 and S2.pdf]

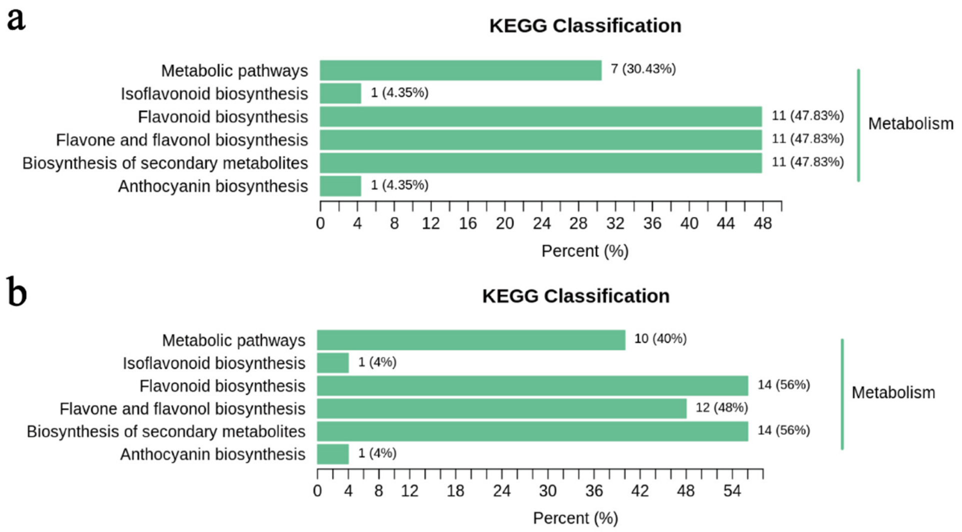

Figure S1. The top 5 KEGG pathways markedly enriched among the DAFs. (**a**) The top 5 pathways enriched in AzLo vs AzLy. (**b**) The top 5 pathways enriched in AoLw vs AoLp.

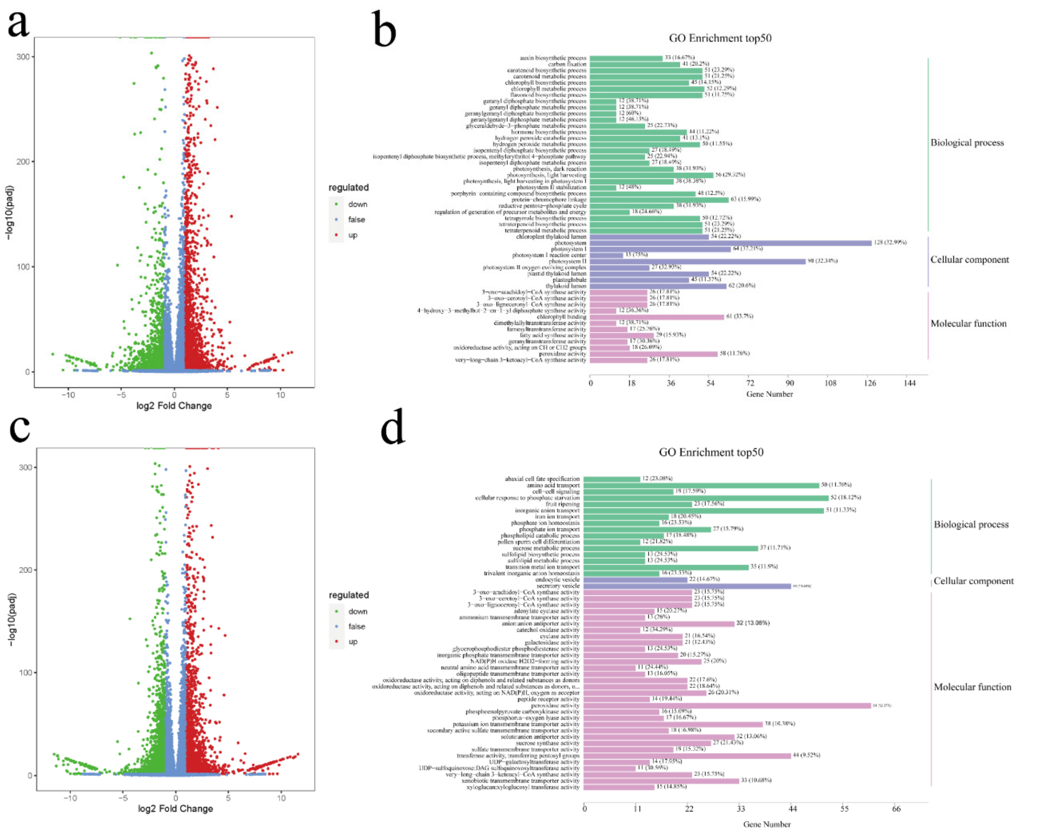

Figure S2. Analysis of DEGs in labellum libraries of *A. zerumbet* and *A. oxyphylla*. (**a**) Volcano plot of DEGs in AzLo vs AzLy; (**b**) GO enriched DEGs in AzLo vs AzLy; (**c**) Volcano plot of DEGs in AoLw vs AoLp; (**d**) GO enriched DEGs in AoLw vs AoLp.
